# Supplementary material for: Responses to SARS-CoV-2 Vaccination in Patients with Cancer (ReCOVer Study): A Prospective Cohort Study of the Hellenic Cooperative Oncology Group
Source: Cancers (Basel). 2021 Sep 15;13(18):4621. doi: 10.3390/cancers13184621 (PMC8466969; doi:10.3390/cancers13184621)

Supplementary Materials

Table S1. Type and number of comorbidities for patients and controls reporting at least one comorbidity at the time of vaccination.

Type of comorbidity	Total (N=120)	Patients (N=98)	Controls (N=22)
COPD	17(14.2)	15(15.3)	2(9.1)
Coronary heart disease	15(12.5)	14(14.3)	1(4.5)
Hypertension	80(66.7)	69(70.4)	11(50.0)
Diabetes	16(13.3)	14(14.3)	2(9.1)
Chronic renal failure	3(2.5)	3(3.1)	0(0.0)
Solid organ transplantation	2(1.7)	1(1.0)	1(4.5)
Blood disease/ disorder	4(3.3)	2(2.0)	2(9.1)
Other	31(25.8)	23(23.5)	8(36.4)
**N of comorbidities**			
1	81(67.5)	64(65.3)	17(77.3)
2	31(25.8)	26(26.5)	5(22.7)
3	7(5.8)	7(7.1)	0(0.0)
4	1(0.83)	1(1.0)	0(0.0)

COPD, Chronic Obstructive Pulmonary Disease; N, number
Percentages were calculated out of the total number of cases experiencing comorbidities.

Figure S1. Anti-SARS-CoV-2 spike IgG values antibodies by cancer type.

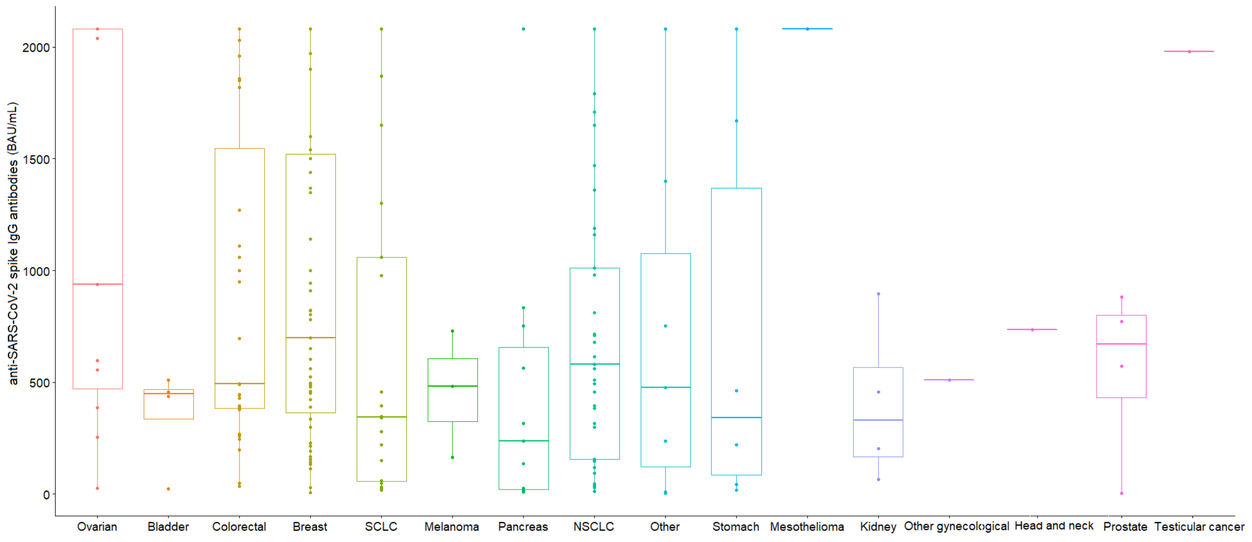

Supplement: Supplementary file 1 [file cancers-13-04621-s001.zip › cancers-1367847-supplementary.pdf]
